# National data opt out programme: consequences for maternity statistics in England

**DOI:** 10.23889/ijpds.v5i1.1126

**Published:** 2020-01-30

**Authors:** KM Lewis, P Hardelid

**Affiliations:** 1 UCL Great Ormond Street Institute of Child Health, London, UK

## Abstract

Electronic health records offer great potential for individual care, service improvement and, when collated, the health of the wider population. Datasets composed of these types of records have been invaluable to our understanding of risk factors for maternal and infant ill-health. However, a potential barrier to data quality in England is emerging where patients choose to opt out of sharing their information beyond the NHS. Focussing on maternity statistics, we will present the importance of population level health data for monitoring NHS services, and the potential consequences for patients of opting out. Evidencing the success of similar systems in Nordic countries, we argue that the English population must be better informed of the implications of opting out of sharing NHS data for research and the safeguards in place to protect patient information.

## Background

Patient records across healthcare providers in England are being digitalised—a process whereby clinical data are stored in digital form and shared with authorised users—to the benefit of patients [1]. When compared to handwritten notes, computerised records improve the detail, completeness and reliability of patient data [1]. Electronic patient record systems improve communication between health professionals within and between different providers [2]. Patients who access their health records online report improved self-care, greater satisfaction with the communication from their doctor and, in some instances, improved safety through patient identification of medication errors [2]. The benefits of electronic health records are not limited to the care of individual patients. When collated, these data are a cost effective way to advance the health of the population through improved knowledge of healthcare services and the aetiologies and treatments of health conditions [3, 4]. Electronic health records in the UK have been successfully used, for example, to investigate: neonatal impact of antibiotic prescription during pregnancy; success of different interpregnancy intervals on pregnancy following miscarriage; and pregnancy complications after caesarean section at first birth [5-7].

However, just as the use of electronic health for research and planning becomes more common place [8], new barriers to the quality of the data are also surfacing. In particular, some patients are choosing not to share their information beyond the NHS for anything other than their direct care. Opting out (see Piel et al. [9] for details about the different types of opt outs) was first made available for patients of the English NHS in January 2014 in response to a recommendation by Dame Fiona Caldicott in her 2013 information governance review [10]. This review was published amid severe concerns, voiced by both the general public and experts in the medical field, about the security of patient data following the release of individual-level health records to profit-making companies [11, 12]. A new consent model intended to be simpler and easier to access (through an online platform) launched in May 2018; however, initial figures suggest that very few patients know about this scheme [13]. As stated in the 2016 Caldicott review [14], ‘patients have a right under the NHS Constitution to request that their personal confidential information is not used beyond their direct care’. We argue that, in parallel with information about this choice, it is imperative that patients have complete and transparent information about the uses and potential advantages of sharing their information. 

## NHS data sharing and safeguards

The data capture organisation within the English NHS, NHS Digital, collects and stores some of the information recorded when individuals receive health or social care in England. This includes records of diagnoses and operations recorded during hospital admissions. The data are used for a variety of reasons including planning NHS services and monitoring patient safety [15]. Strictly controlled release of some patient information may be shared with NHS providers and commissioners, university researchers, charities and companies that are partnered with the NHS. Where permission is granted, all organisations must follow stringent protocols when storing and analysing the data. Personal identifiers, such as names and NHS numbers are removed in all circumstances apart from where specific patient consent is given or where required by the law. NHS Digital states that ‘we make sure data is only used for the good of health and care’, and all organisations go through a lengthy application process to ensure this is the case.

## NHS maternity statistics

The most comprehensive source of information on all births and deliveries in the NHS in England is Hospital Episode Statistics (HES); a dataset that includes all admissions to NHS or NHS-funded hospitals in England [3]. Delivery information, such as the place of delivery, baby’s sex, birthweight, gestational age and method of delivery, is used for many purposes, including to create resources for parents-to-be, to evaluate and improve maternity care provision and to investigate multiple risk factors for ill-health in mothers and babies [16]. Tools have been created utilising this data to aid expectant parents when making maternity choices. One such resource, the Birth Choices tool from Which? [17] recommends considering essential maternity statistics when planning which hospital to give birth in, such as variation in caesarean sections, induction and other medical interventions. Further, the National Maternity and Perinatal Audit (NMPA) was set up in 2016 to evaluate quality in NHS maternity services [18]. Using maternity data from HES linked with data from each maternity unit, the NMPA provides a range of statistics comparing outcomes at maternity unit level, including induction of labour and caesarean section rates. In addition, HES has been used for maternal and child health research to examine, for example, factors explaining excess child mortality in England and the safety of surgical procedures during pregnancy [19, 20]. Individual level data are necessary for this type of research, since this allows multiple risk factors for maternal and child outcomes to be taken into account.

## Opting out and the effect on the quality of NHS maternity data

**Figure 1: Rates of patients opting out, by CCG: England (with inlay map of London CCGs), as at December 2018. Data from NHS Digital [21]:**
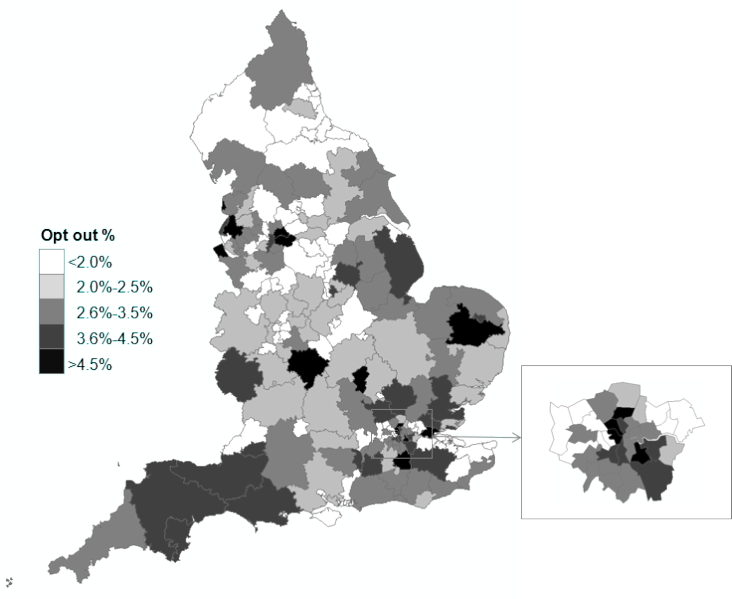


As at 1 December 2018, the average national data opt out rate across England was 2.8% [21]. Top-level demographic information published by NHS Digital shows that rates of opt outs are higher in older people and females [21]. This is in keeping with findings from surveys and qualitative research that the characteristics of people who are less willing to share their health data with researchers differ from those who are willing to consent (although these characteristics have not been consistent across studies) [22, 23]. For example, a study of mothers in the UK Millennium Cohort Study found that the proportion consenting to link survey data with their child’s NHS records differed by country of residence, age, ethnicity, lone parenthood status and education [24]. As shown in Figure 1, opt out rates in the general population are also not uniformly distributed across geographical area in England. Twelve Clinical Commissioning Groups (CCGs) have opt out rates higher than 5% and, strikingly, one CCG has a rate of 10.1% [21]. At the GP practice level, there are instances where the entire patient population have opted out [21]. As remarked by Piel et al. [9], this raises significant questions about whether the patients in these practices explicitly opted out for themselves.

To exemplify the impact of biased data on maternal information, we simulated outcome rates for two health indicators had opt outs not been applied to the data. We downloaded publically available CCG-level information from Public Health England’s Fingertips Child and Maternal Health Profiles for one common and one rare outcome, the proportion of deliveries with caesarean sections (occurring in approximately 27% of births nationwide) and births with very low birth weight (<1500g, 1.2% nationwide) [25]. We chose three CCGs for our example, each with a different rate of opt out, as published at 1st December 2018 by NHS Digital [26]. Under the assumption that the women in the maternity dataset opted out at the same rate as the whole CCG population, we modelled three scenarios based on the rate of events in the women that opted out: 1) no events; 2) events occurring at the same rate as women who did not opt out; 3) all had an event. Mirroring methods used by Public Health England [27], 95% confidence intervals (CIs) were calculated in Stata 15.0 [28] using the Wilson Score method. Microsoft Excel 2013 was used to create graphs.

**Table 1: Published and simulated number of events, patients and event rates, by indicator and CCG. Data from the National Child and Maternal Health Intelligence Network [25] and NHS Digital [26]:** *Scenario 1 (no new events) = published events/(deliveries + extra deliveries) where extra deliveries= published deliveries/(1-opt out rate)-published deliveries; scenario 2 (average events) = ((extra deliveries*event rate)+published events)/(deliveries + extra deliveries); scenario 3 (max. events) = (events+ extra deliveries)/(deliveries + extra deliveries).

	Caesarean section			Very low birth weight		
	Bradford City CCG	Merton CCG	Oldham CCG	Bradford City CCG	Merton CCG	Oldham CCG
CCG opt out rate (%) [26]	0.3	2.74	10.08	0.3	2.74	10.08

Published maternity data [25]					

Events	478	948	792	36	39	39
Deliveries	2024	3265	2797	1657	3219	3195
Event rate	23.6	29	28.3	2.17	1.21	1.22
(95% CI)	(21.8,25.5)	(27.5,30.6)	(26.7,30.0)	(1.57,2.99)	(0.89,1.65)	(0.89,1.66)

New event rate (95% CI)*					

Scenario (1)	23.5	28.2	25.5	2.17	1.18	1.1
	(21.8,25.4)	(26.7,30.0)	(24.0,27.0)	(1.57,2.99)	(0.86,1.61)	(0.80,1.50)
Scenario (2)	23.6	29	28.3	2.17	1.21	1.2
	(21.8,25.5)	(27.5,30.6)	(26.8,30.0)	(1.57,2.99)	(0.89,1.64)	(0.90,1.63)
Scenario (3)	23.8	31	35.6	2.47	3.93	11.2
	(22.0,25.7)	(29.4,32.6)	(34.0,37.3)	(1.82,3.33)	(3.32,4.64)	(10.2,12.3)

**Figure 2: Observed and simulated rates of deliveries by caesarean section: By CCG, 2016/17 [21, 25]:**
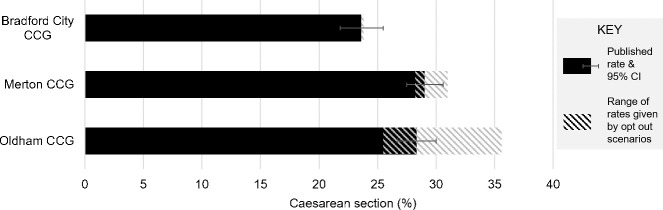


**Figure 3: Observed and simulated rates of births with very low birth weight (<1500g) observed and simulated rates: by CCG, 2016 [21, 25]:**
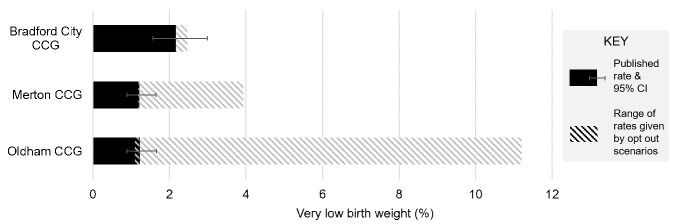


Table 1 displays published and simulated event rates for caesarean sections and infants born with very low birth weight for Oldham (10.1% opt outs), Merton (2.8% opt outs) and Bradford City (0.3% opt outs) CCGs. In scenario 2, where patients opting out have the same rate of events as patients not opting out, published and simulated event rates do not differ. Figure 2 and 3 show the possible range of rates between the extremes of scenario 1 (no new events) and scenario 3 (all opt outs have an event). These scenarios show that both the CCGs with average (Merton) and high (Oldham) rates of opting out could be showing misleading information. For the rarer event, births with very low birth weight, the rate of births with this outcome could potentially be 9-fold higher than the published rate.

The non-random nature of opt-outs has potentially large implications for the outcomes of public health monitoring, research studies and clinical audits. At an NHS trust level, information bias could produce flawed outputs in audits intending to suggest improvements and highlighting good practice. These biases will also affect the reliability of findings on maternal and children’s health research. As shown in our simulation, this is particularly the case where less common (but often more serious) outcomes are studied. Problematically, the bias introduced into datasets with opt outs applied cannot be treated with the same statistical methods used to treat missing data. Multiple imputation, a method which is commonly applied to deal with missing data, relies on the assumption that the missing information can be explained by differences in the observed data [29]. However, once patients have opted out, their data is completely removed from the dataset (i.e. complete case removal) meaning that we cannot account for systematic differences. Using multiple imputation in these instances may actually add further bias to results [29]. Methods used in population-based surveys to overcome biases of non-consent, such as weighting adjustments or simulation studies ideally require detailed data on the population (e.g. by gender, age, deprivation level and local area) who have opted out so that correct weights can be derived - currently not published by NHS Digital. Research to determine whether those who have opted out of sharing data are different in terms of socio-demographic and health characteristics, in specific population such as expectant mothers, would help in applying such methods to tackle data missing due to opt outs. 

## An inevitable problem?

The experience of public health researchers in the Nordic countries demonstrate that data sharing can be achieved with buy-in from citizens and at great value to clinical research [30]. In these countries, residents are assigned a personal identity number from birth, which can be used to track individuals across time and generations. Unlike the UK equivalent (NHS number), which is used only in health and social care settings [31], personal identity numbers are used across a multitude of sectors. This means that individual data can be linked across health, education and social security datasets, for example, providing high quality comprehensive information on risk factors for ill-health and other outcomes. Research using these administrative datasets have contributed markedly to the evidence base regarding determinants of health and disease across the life-course. Valuable research outcomes include the long-term social and medical consequences of preterm birth and heritability of pre-eclampsia, amongst others [32, 33].

Before a data-based research project can begin, approval must be gained from a regional or national ethics committee. To protect confidentiality, personal identifiers are not shared with researchers and results cannot be published at an individual level [34]. In essence, the safeguards in place are very similar to those in England. In contrast, however, there is broad public general awareness and acceptance of the use of individual data in research and a long-standing culture of trust in public services and data donation for the good of the population [4, 30]. More research is needed on why this discrepancy in public perception of using administrative data for research between England and the Nordic countries has arisen, what can be done to improve public trust in England, and who is best placed to do it.

Evidence from England suggests that greater knowledge of research processes and safeguards improves the likelihood of acceptance of electronic health records being used without explicit consent [35]. An electronic real-time dataset integrating primary and secondary care was successfully implemented over a decade ago in Salford, Manchester. All patients were sent a letter with information and a query about opting out. Less than 0.2% of the nearly quarter of a million patients chose to opt out [36]. In contrast, information about the now withdrawn care.data scheme to integrate primary and secondary care records was disseminated by generic leaflets, which were reportedly not seen by the majority of the population [11]. However, given that some of the opt-outs may have been driven by GP practices rather than patient-level decisions [9], it is not clear to what extent whether it was the NHS information campaign directed at patients (or indeed lack of it) that led to the 2.8% opt-out rate, or a lack of buy-in from clinicians.

In terms of providing information to the general public, some lessons have been learnt since care.data. A national radio campaign ran for 6-weeks after the launch of the new opt out system and NHS Digital’s website now links to Understanding Patient Data [15], an informative website run by Wellcome Trust. To further exhibit the benefits of sharing data for audits and research, NHS Digital could start by listing examples of how health care data have been used for research, as is available in other NHS held datasets (e.g. CPRD [37]). Other innovative examples of dissemination include the University of Manchester’s citizen’s jury on health records [38] and an animation created as part of the #datasaveslives campaign by the Farr Institute [39]. However, the impact and wider reach of these schemes are not clear.

Arguably, information about the benefit of data sharing can only go so far in raising public confidence. Evidence from the research literature and reflected in media coverage suggests that there is unease about the potential of commercial entities, such as pharmaceutical and insurance companies, to make profit from NHS data. This is in contrast to the largely positive view of university researchers or NHS staff making use of this data [22, 36]. Therefore, Wellcome Trust’s call for clear examples of ‘acceptable and unacceptable purposes’ for which data can and cannot be used, amongst other steps, should be heeded [15]. NHS Digital is beginning to advertise the benefits of sharing NHS data for research and planning purposes, and it is vital these efforts are continued and extended. Clinicians play a pivotal role in the discourse of patient consent to use NHS data for research. Their concerns must be better understood and addressed in future consultations and information campaigns about using data for research.

## Conclusion

When patients to choose to opt out of sharing data beyond their direct care, the reliability of service information and evaluation and wider research based on electronic health records is diminished. We call for more transparent, clear and detailed information on: who can apply to use NHS data and for what reasons; the safeguards in place to protect individual information; and, importantly, the wider consequences of opting out on population health research and public health service information. Only with this information can individuals be expected make an informed decision about opting out of sharing their data.

## Acknowledgments

Patient involvement: We thank Maurice Hoffman, Shilpa Patel, Katherine Ruane and other representatives from the Farr Institute London Public Panel for their perspectives on the issues discussed, as well as specific comments on an earlier version of this paper.

## Ethics Statement

This study used open source aggregate-level data and, therefore, ethical approval was not required.

## Abbreviations

CCG	Clinical Commissioning Group
HES	Hospital Episode Statistics
